# Barcelona Scoliosis Physical Therapy School – BSPTS – based on classical Schroth principles: short term effects on back asymmetry in idiopathic scoliosis

**DOI:** 10.1186/1748-7161-7-S1-O57

**Published:** 2012-01-27

**Authors:** M Jelačić, M Villagrasa, E Pou, G Quera-Salvá, M Rigo

**Affiliations:** 1 Institut Elena Salva, Barcelona, Spain

## Background

Previous results have shown the specificity of Schroth exercises (according to BSPTS protocol) but in a series including patients under bracing [1-4].

## Objective

To investigate the short term effects of an intensive program of exercises on back asymmetry in idiopathic scoliosis with no other treatment.

## Materials and methods

Retrospective, including 47 patients with IS treated exclusively with exercises. Mean age 18.64 ± 5.78 years. Outpatient Intensive Rehabilitation was carried out, three hours a day, five days a week, 4 weeks. Surface topography (Formetric) was performed to measure trunk imbalance, surface rotation and lateral deviation before and after the treatment period. The obtained pre- and post-treatment values were then compared.

## Results

The mean trunk imbalance prior to and after the treatment was 10.16 mm and 8.53 mm respectively (p<0.05). The pre-treatment mean value of the lateral deviation (rms) was 13.92 mm, compared to the post-treatment one of 11.96 mm (p<0.05) and of the lateral deviation (max) was 25.6 mm and 21.42 mm respectively (p<0.05). The mean initial value of the surface rotation (rms) was 6.88 degrees, reaching 6.52 degrees at the end of the treatment (p<0.05) and of the surface rotation (max) 13.22 degrees and 11.88 degrees respectively (p<0.05).

## Conclusions

Current results suggest that exercises according to Schroth principles, following BSPTS protocol, are able to improve back asymmetry, spinal imbalance in the frontal plane and virtual spinal geometry in a short term, confirming specificity in its mechanics of action.
